# Data on the flexural vibration of thin plate with elastically restrained edges: Finite element method and wave based method simulations

**DOI:** 10.1016/j.dib.2020.105883

**Published:** 2020-06-29

**Authors:** Ling Liu, Roberto Corradi, Francesco Ripamonti, Zhushi Rao

**Affiliations:** a State Key Laboratory of Mechanical System and Vibration, School of Mechanical Engineering, Shanghai Jiao Tong University, Shanghai 200240, China; b Department of Mechanical Engineering, Politecnico di Milano, Milano 20156, Italy

**Keywords:** Wave based method, Elastically restrained edge, Damping connection, Non-uniform boundary conditions, Kirchhoff plate, Rectangular plate, Pentagonal plate, Plate bending problem

## Abstract

The data here reported refer to the numerical examples shown in the research article “Wave based method for flexural vibration of thin plate with general elastically restrained edges” (Liu et al., 2020 [1]). Within the examples, only the datasets regarding the plates with elastic or elastic-damping supports are provided. The datasets contain the raw data directly obtained from the forced vibration simulations. The simulations are carried out using two methods: the finite element method realized in ANSYS Mechanical APDL and the proposed wave based method (Liu et al., 2020 [1]), implemented in a MATLAB code. The data obtained from ANSYS serves as reference for the response of the plate under different boundary conditions. For each frequency, the transverse displacements of the plate at two pre-selected points are listed in the spreadsheet (e.g. MS Excel). When damping is present, they are separated into real part and imaginary part. This part of data can be used as reference when other novel methods are developed. The datasets obtained from MATLAB include the contribution factors as well as the wave functions. Based on them, one can obtain the displacement as a complex number at any point of the plate after a simple postprocessing. Postprocessing codes to obtain the frequency response function for a user-given point and the displacement field at a user-given frequency are also provided. This part of data presents much more information than the previous part as well as the corresponding results in the related research article. It makes it possible to see the responses at other points or other frequencies that are not considered in the research article, without repeating the time-consuming simulations. Moreover, if someone wants to further improve the wave based method, this part of data will be helpful, either for analysing the limitations of the proposed method or for more direct comparisons. Any research related to the flexural vibration of plate can also consider the data provided in this article.

**Specifications Table**

**Table:** 

**Subject**	Mechanical Engineering
**Specific subject area**	Structural vibration
**Type of data**	Table (excel files) Dataset (mat files) Postprocessing codes (m files)
**How data were acquired**	Data in the excel files were obtained through the simulations in ANSYS Mechanical APDL 17.2, using Finite Element Method (FEM). Datasets in the mat files were obtained from the numerical computation implemented in MATLAB, using the modified Wave Based Method (WBM) introduced in the associated research article [1].
**Data format**	Raw
**Parameters for data collection**	In the numerical simulations of the plate, the important parameters include geometric parameters, material parameters, excitation parameters, boundary conditions, measuring points and quantity, and modelling parameters. While the modelling parameters refer to element type and size in FEM, they are wave functions and truncation factors in WBM.
**Description of data collection**	The datasets were collected from five numerical examples. They respectively analyse the rectangular plate with uniformly elastic supports (E-E-E-E), with uniformly elastic-damping supports (ED-ED-ED-ED), with non-uniformly elastic supports (E1-E2-E3-E4), with elastic-damping partially supports (PS), and the irregular pentagonal plate with non-uniformly elastic-damping supports (IR). The E-E-E-E example considers five cases regarding different spring constants. The ED-ED-ED-ED example has two cases for different damping coefficients.
**Data source location**	Institution: Department of Mechanical Engineering, Politecnico di Milano City/Town/Region: Milan Country: Italy
**Data accessibility**	With the article
**Related research article**	L. Liu, R. Corradi, F. Ripamonti, Z. Rao, Wave based method for flexural vibration of thin plate with general elastically restrained edges, J. Sound Vib. In Press.

**Value of the data**

• The data is useful for investigating the flexural vibration of thin plates, developing new numerical methods for structural dynamic problems and improving the wave based method.
• The data is useful for engineers and researchers who need to control or analyse the vibration of a structure like plate, and also helpful for those who work on the numerical prediction tools.
• When developing other methods for plate bending problem, one can use the data for comparison or as reference.
• The data can be further analysed to get more insight in the flexural vibration of thin plates. For example, it is possible to investigate the influence of boundary conditions by additional comparison among datasets.
• The researchers interested in the WBM might find the postprocessing codes useful. Working on the WBM for plate bending problems, they can directly get the wave functions and the solutions of contribution factors.

## 1 Data description

**Fig. 1:**
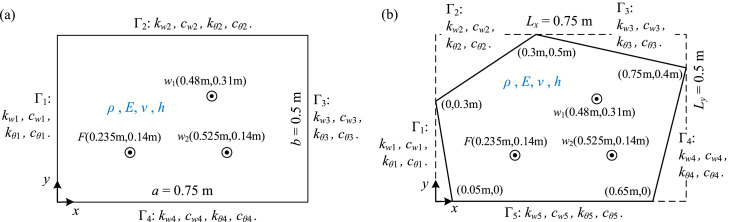
The plates with general elastically restrained edges: (a) the rectangular plate for numerical examples E–E–E–E, ED–ED–ED–ED, E1–E2–E3–E4 and PS; (b) the irregularly-shaped pentagonal plate for numerical example IR.

The data presented in this article were obtained from the numerical simulations of two plates with general elastically restrained edges. The two plates are shown in Fig. 1. They are made of the same material with density $\rho =2700\text{kg}/m^{3}$, Young's modulus $E=70\times 10^{9}N/m^{2}$, and Poisson's ratio ν=0.3. They also have the same thickness h=0.002m. The edges of the plates are restrained by springs and dampers. The transverse displacement of edge Γ*_i_* is subject to the linear spring with stiffness *k_wi_*, and the linear damper with damping coefficient *c_wi_*. Meanwhile, the rotational displacement of edge Γ*_i_* is subject to the rotational spring with stiffness *k_θi_*, and the rotational damper with the damping coefficient *c_θi_*. All the stiffnesses and damping coefficients are defined per unit length, and the rotational springs and dampers act in the direction aligned with the bending moment. For the two plates, the simulations evaluate their flexural vibration when they are under the steady-state force excitation *e^jωt^* at point *F*(0.235m, 0.14m). The amplitude of the force is 1 N and *ω* is the circular frequency.

As shown in Fig. 1(a), the first plate is rectangular with the dimensions a×b=0.75m×0.5m. Datasets include the results of this plate under four types of boundary conditions (BCs) with respect to the supports along edges, i.e., the uniformly elastic supports (E–E–E–E), the uniformly elastic-damping supports (ED-ED-ED-ED), the non-uniformly elastic supports (E1–E2–E3–E4) and the elastic-damping partially supports (PS). Among them, the E-E-E-E type contains five cases, with respect to the different combinations of translational stiffness *k_w_* and rotational stiffness *k_θ_*. The ED-ED-ED-ED type has two cases, corresponding to the two levels of damping, $c_{w}/k_{w}=c_{\theta }/k_{\theta }=0.001$ and $c_{w}/k_{w}=c_{\theta }/k_{\theta }=0.01$.

The second plate is pentagonal, and its geometry is shown in Fig. 1(b), determined by the coordinates of its vertexes. As is shown, the pentagonal plate can be enclosed by the rectangular with dimensions $L_{x}\times L_{y}=0.75m\times 0.5m$. Regarding this plate, only the datasets for one type of BCs are provided. In this case, the plate is with the non-uniformly elastic-damping supports (ED1-ED2–ED3–ED4–ED5). Since it is the only case for an irregularly-shaped plate, it is represented in short using “IR” and the corresponding datasets were named with “IR”.

For all the simulations, datasets are provided separately for the two different numerical methods. Data collected from ANSYS FEM are the transverse displacements of the points *w*_1_ and *w*_2_ (see Fig. 1) at each computed frequency. In no-damping cases, only the real part is provided. In the damping cases, the real part and imaginary part are separated into two columns. The corresponding cases, as well as the available frequency range for each excel file, are specified in Table 1. The stiffnesses are given as their non-dimensional forms and the damping coefficients are related to the stiffnesses. The notations can refer to Fig. 1, and *D* is the bending stiffness of the plate, which is given by $D=Eh^{3}/[12\left(1-\nu^{2}\right)]$.

**Table 1:** Summary of the simulation cases corresponding to the datasets collected from ANSYS FEM. In these excel files are the tables for the transverse displacements of the points *w*_1_ and *w*_2_ (shown in Fig. 1) within the given frequency range at the intervals of 1 Hz.

Excel filename	Sheet	Tyre of BCs	Stiffness	Damping $c_{w}/k_{w}=c_{\theta }/k_{\theta }$	Frequency (Hz)
**ANSYS_EEEE**	Kw100Ka1000	E–E–E–E	$k_{w}a^{3}/D=100,k_{\theta }a/D=1000$	0	1–1000
	Kw10Ka1000	E–E–E–E	$k_{w}a^{3}/D=10,k_{\theta }a/D=1000$	0	200–500
	Kw1000Ka1000	E–E–E–E	$k_{w}a^{3}/D=1000,k_{\theta }a/D=1000$	0	200–500
	Kw100Ka10	E–E–E–E	$k_{w}a^{3}/D=100,k_{\theta }a/D=10$	0	200–500
	Kw100Ka100	E–E–E–E	$k_{w}a^{3}/D=100,k_{\theta }a/D=100$	0	200–500
**ANSYS_EDEDEDED**	C0001	ED–ED–ED–ED	$k_{w}a^{3}/D=100,k_{\theta }a/D=1000$	0.001	200–500
	C001	ED–ED–ED–ED	$k_{w}a^{3}/D=100,k_{\theta }a/D=1000$	0.01	200–500
**ANSYS_E1E2E3E4**	C=0	E1–E2–E3–E4	$k_{w1}a^{3}/D=k_{\theta 1}a/D=10$ $k_{w2}a^{3}/D=k_{\theta 2}a/D=100$ $k_{w3}a^{3}/D=k_{\theta 3}a/D=1000$ $k_{w4}a^{3}/D=k_{\theta 4}a/D=10000$	0	200–500
**ANSYS_PS**	C0001	PS	$k_{w}a^{3}/D=100,k_{\theta }a/D=1000$*	0.001*	200–500
**ANSYS_IR**	C0001	IR (ED1–ED2–ED3–ED4–ED5)	$k_{w1}L_{x}^{3}/D=10^{4},k_{\theta 1}L_{x}/D=10^{2}$ $k_{w2}L_{x}^{3}/D=10^{3},k_{\theta 2}L_{x}/D=10^{4}$ $k_{w3}L_{x}^{3}/D=10^{4},k_{\theta 3}L_{x}/D=10^{3}$ $k_{w4}L_{x}^{3}/D=10^{3},k_{\theta 4}L_{x}/D=10^{2}$ $k_{w5}L_{x}^{3}/D=10^{2},k_{\theta 5}L_{x}/D=10^{3}$	0.001	200–500

⁎ For the PS type, the stiffness and damping parameters are only for the supported parts, i.e., edges from *a*/4 to 3*a*/4 in *x* direction and from *b*/4 to 3*b*/4 in *y* direction. Otherwise, the values are zero.

Data obtained from MATLAB WBM are the contribution factors of wave functions, which are the primary solutions of the wave based model. The datasets are saved in the MATLAB workspace (mat files). The corresponding cases of these mat files, as well as the available frequency range are summarized in Table 2. Since the truncation factor *T*, which are used to limit the number of wave functions, is not always the same for all cases, its value is also listed in Table 2 for each case. For the case of the pentagonal plate (IR), two datasets are provided. The dataset “PDD_IR_T_2” was obtained with T=2, and “PDD_IR_T_2” was obtained with T=4. The latter one, with larger value of *T*, used more wave functions in the simulation and provides more accurate results. Each mat file in Table 2 is the dataset for a single simulation. It contains the information that is necessary for postprocessing and specific to the corresponding simulation. There are three variables. One is the structure array “PDD”, which contains two fields: “PDD.freq” is for the frequency and “PDD.coe” is for the contribution factors. For each frequency, the contribution factors are expressed as a vector in the “PDD.coe”. The other two variables are the flag for damping “D_flag” and the truncation factor *T*. If damping is considered in the BCs, “D_flag” is equal to 1, otherwise “D_flag” is equal to 0. The truncation factor *T* is a parameter that is used to limit the number of wave functions based on the truncation rule [1,2]:

(1) $$\frac{n_{S_{1}}\pi }{L_{x}}\approx \frac{n_{S_{2}}\pi }{L_{y}}\geq Tk_{b}.$$

where, *k_b_* is the plate bending wavenumber and $k_{b}=\sqrt[{4}]{\rho h\omega^{2}/D}$, $n_{s_{1}}\pi /L_{x}\left(n_{s1}\in N\right)$ and $n_{s_{2}}\pi /L_{y}\left(n_{s2}\in N\right)$ are the largest wavenumbers of the considered wave functions. In other words, the wave functions with the wavenumbers $k_{xs_{1}}=s_{1}\pi /L_{x}$ ($s_{1}=0,1,2,\ldots ,n_{s_{1}}$) and $k_{ys_{2}}=s_{2}\pi /L_{y}$ ($s_{2}=0,1,2,\ldots ,n_{s_{2}}$) are used for the wave based modelling. Then, the total number of wave functions is given by $n_{s}=4\left(n_{s_{1}}+1\right)+4\left(n_{s_{2}}+1\right)$, which also indicates *n_s_* unknown contribution factors of the wave functions. Therefore, the truncation factor *T* in the dataset records the wave functions that are used in the particular simulation.

**Table 2:** Summary of the simulation cases corresponding to the datasets collected from MATLAB WBM. In these mat files are the wave function contribution factors for each computed frequency.

Mat filename	Tyre of BCs	Stiffness	Damping $c_{w}/k_{w}=c_{\theta }/k_{\theta }$	Frequency (Hz)	*T*
**PDD_Kw100_Ka1000**	E–E–E–E	$k_{w}a^{3}/D=100,k_{\theta }a/D=1000$	0	1–1000	2
**PDD_Kw10_Ka1000**	E–E–E–E	$k_{w}a^{3}/D=10,k_{\theta }a/D=1000$	0	200–500	2
**PDD_Kw1000_Ka1000**	E–E–E–E	$k_{w}a^{3}/D=1000,k_{\theta }a/D=1000$	0	200–500	2
**PDD_Kw100_Ka10**	E–E–E–E	$k_{w}a^{3}/D=100,k_{\theta }a/D=10$	0	200–500	2
**PDD_Kw100_Ka100**	E–E–E–E	$k_{w}a^{3}/D=100,k_{\theta }a/D=100$	0	200–500	2
**PDD_ED0001**	ED–ED–ED–ED	$k_{w}a^{3}/D=100,k_{\theta }a/D=1000$	0.001	200–500	2
**PDD_ED001**	ED–ED–ED–ED	$k_{w}a^{3}/D=100,k_{\theta }a/D=1000$	0.01	200–500	2
**PDD_E1E2E3E4**	E1–E2–E3–E4	$k_{w1}a^{3}/D=k_{\theta 1}a/D=10$ $k_{w2}a^{3}/D=k_{\theta 2}a/D=100$ $k_{w3}a^{3}/D=k_{\theta 3}a/D=1000$ $k_{w4}a^{3}/D=k_{\theta 4}a/D=10,000$	0	200–500	2
**PDD_PS_T_4**	PS	$k_{w}a^{3}/D=100,k_{\theta }a/D=1000$*	0.001*	200–500	4
**PDD_IR_T_2, PDD_IR_T_4**	IR (ED1–ED2–ED3–ED4–ED5)	$k_{w1}L_{x}^{3}/D=10^{4},k_{\theta 1}L_{x}/D=10^{2}$ $k_{w2}L_{x}^{3}/D=10^{3},k_{\theta 2}L_{x}/D=10^{4}$ $k_{w3}L_{x}^{3}/D=10^{4},k_{\theta 3}L_{x}/D=10^{3}$ $k_{w4}L_{x}^{3}/D=10^{3},k_{\theta 4}L_{x}/D=10^{2}$ $k_{w5}L_{x}^{3}/D=10^{2},k_{\theta 5}L_{x}/D=10^{3}$	0.001	200–500	2 or 4

⁎ For the PS type, the stiffness and damping parameters are only for the supported parts, i.e., edges from *a*/4 to 3*a*/4 in *x* direction and from *b*/4 to 3*b*/4 in *y* direction. Otherwise, the values are zero.

For the WBM, postprocessing is necessary to get the plate displacement. Hence, postprocessing codes are provided. With these codes and the mat files, more results than those shown in the associated research article can be obtained. There are five MATLAB codes (m files) in the supplementary files:

• “Postprocessing_FRF.m” is to obtain the Frequency Response Function (receptance) at any point of the plates;
• “Postprocessing_Field.m” is to get the displacement field for the rectangular plate;
• “Postprocessing_Field_IR.m” is to get the displacement field for the pentagonal plate;
• “CoeBaseFuncX.m” and “CoeBaseFuncY.m” are the functions used to construct the wave functions in the postprocessing m files, so that the contribution factors can combine with the wave functions to obtain the displacements.

There is also an additional mat file (map.mat) containing the colour information for the contour plots in the “Postprocessing_Field.m” and “Postprocessing_Field.m”.

## 2 Experimental design, materials and methods

Numerical simulations were carried out on two thin plates that satisfy the Kirchhoff plate theory [3]. The two plates have different shapes and plane sizes, as shown in Fig. 1, but the same thickness h=0.002m. Both plates are made of aluminium. The material parameters $\rho =2700\text{kg}/m^{3}$, $E=70\times 10^{9}N/m^{2}$ and ν=0.3. A steady state excitation through a unit normal force acting on point *F*(0.235m, 0.14m) is considered. The translational and rotational displacements of the plate along edges were restrained with elasticity and damping. The restraints were modelled using translational and rotational springs and dampers. Translational and rotational spring constants were respectively given by *k_w_* and *k_θ_*, and the corresponding damping coefficients were given by *c_w_* and *c_θ_*. The boundary conditions were determined by their values and, for different simulations, the values are shown in Tables 1 and 2. The numerical methods for simulations are the FEM achieved via ANSYS Mechanical APDL 17.2 and the WBM implemented in MATLAB.

For the simulations in ANSYS, the two plates were modelled by SHELL63, the four-node linear shell element based on the Kirchhoff-Love theory, with six degrees of freedom at each node. The unused degrees of freedom, i.e. the translations in *x* and *y* directions and the rotation in *z* direction, were set to zero. Element size was 0.0025 m, for the rectangular plate, and 0.0024 m, for the pentagonal plate. Along the edges of the plates, the generic elastically restraints were simulated using COMBIN14, the nodal-based spring-damper element. Each COMBIN14 element combined one fixed node and one node on the plate edge, with a single degree of freedom, either translation in the *z* direction or rotation along the edge. Since the edge pressure is allocated to the element nodes, the continuous stiffness and damping along the edge should also be allocated to nodes. For the length *l_e_* between two nodes, the stiffness and damping allocated to the two nodes were given by

(2) $$k^{+}=k^{-}=\frac{kl_{e}}{2},\;c^{+}=c^{-}=\frac{cl_{e}}{2},$$

where ‘+’ denotes the left side of the length *l_e_* and ‘–’ denotes the right side, *k* and *c* are respectively the stiffness and damping per unit length. Each of the nodes along the restrained edges are connected to one COMBIN14 element for translation and one COMBIN14 element for rotation. After applying the unit force at the point *F*(0.235m, 0.14m), harmonic analysis was performed at the intervals of 1 Hz within the frequency range listed in Table 1, for each corresponding simulation case. After the simulations, the displacement at points *w*_1_(0.48m, 0.31m) and *w*_2_(0.525m, 0.14m) in terms of its real part and imaginary part were listed and saved to the corresponding spreadsheet (excel files).

For the simulations in MATLAB, they followed the WBM for flexural vibration of thin plate with general elastically restrained edges proposed in the associated research article [1]. The method is modified from the conventional WBM [4] for plate with classical boundary conditions. After defining the geometry, material properties, excitation force and boundary conditions, the wave based model was built for each computing frequency. The main points in the modelling process are the definition of wave functions, the particular solution of the force and the implementation of the weighted residual formulation. Meanwhile, the truncation rule Eq. (1) is used to limit the degrees of freedom of the model, which is equal to the number of wave functions. Based on Eq. (1), more wave functions will be used if the truncation factor *T* is increased. Since the wave based model converges towards the exact solution with the increase of the degrees of freedom [3], the truncation factor *T* is also related to the computation accuracy. In practice, T=2 was the start point and then *T* was increased to check the convergence. For the integrations in the weighted residual formulation, Gauss-Legendre quadrature was applied, and 51 Guass points were used for each edge. The set-up model is a linear algebraic equation system. Solution of the equation system is a vector of the wave function contribution factors. With the contribution factors stored in the database, the modelling and solution processes were repeated for next frequency. After finishing the solutions of all the frequencies for one case, the dataset was saved to the corresponding mat file, as shown in Table 2.

## Declaration of Competing Interest

The authors declare that they have no known competing financial interests or personal relationships which have, or could be perceived to have, influenced the work reported in this article.

## Appendix Supplementary materials

Image, application 1
